# *De Novo* RNA Sequencing and Transcriptome Analysis of *Colletotrichum gloeosporioides* ES026 Reveal Genes Related to Biosynthesis of Huperzine A

**DOI:** 10.1371/journal.pone.0120809

**Published:** 2015-03-23

**Authors:** Guowei Zhang, Wenjuan Wang, Xiangmei Zhang, Qianqian Xia, Xinmei Zhao, Youngjoon Ahn, Nevin Ahmed, Andreea Cosoveanu, Mo Wang, Jialu Wang, Shaohua Shu

**Affiliations:** 1 College of Plant Science & Technology, Huazhong Agricultural University, Wuhan, China; 2 College of Life Sciences, Xinyang Normal University, Xinyang, China; 3 College of Agriculture and Life Sciences, Seoul National University, Seoul 151–742, Republic of Korea; 4 Department of Plant Protection, Faculty of Agriculture, Benha University, Benha, Egypt; 5 Phytopathology Unit, Faculty of Biology, University of La Laguna, La Laguna, Tenerife, Spain; University of New South Wales, AUSTRALIA

## Abstract

Huperzine A is important in the treatment of Alzheimer’s disease. There are major challenges for the mass production of huperzine A from plants due to the limited number of huperzine-A-producing plants, as well as the low content of huperzine A in these plants. Various endophytic fungi produce huperzine A. *Colletotrichum gloeosporioides* ES026 was previously isolated from a huperzine-A-producing plant *Huperzia serrata*, and this fungus also produces huperzine A. In this study, *de novo* RNA sequencing of *C*. *gloeosporioides* ES026 was carried out with an Illumina HiSeq2000. A total of 4,324,299,051 bp from 50,442,617 high-quality sequence reads of ES026 were obtained. These raw data were assembled into 24,998 unigenes, 40,536,684 residues and 19,790 genes. The majority of the unique sequences were assigned to corresponding putative functions based on BLAST searches of public databases. The molecular functions, biological processes and biochemical pathways of these unique sequences were determined using gene ontology (GO) and Kyoto Encyclopedia of Genes and Genomes (KEGG) assignments. A gene encoding copper amine oxidase (CAO) (unigene 9322) was annotated for the conversion of cadaverine to 5-aminopentanal in the biosynthesis of huperzine A. This gene was also detected in the root, stem and leaf of *H*. *serrata*. Furthermore, a close relationship was observed between expression of the CAO gene (unigene 9322) and quantity of crude huperzine A extracted from ES026. Therefore, CAO might be involved in the biosynthesis of huperzine A and it most likely plays a key role in regulating the content of huperzine A in ES026.

## Introduction

**Fig 1:**
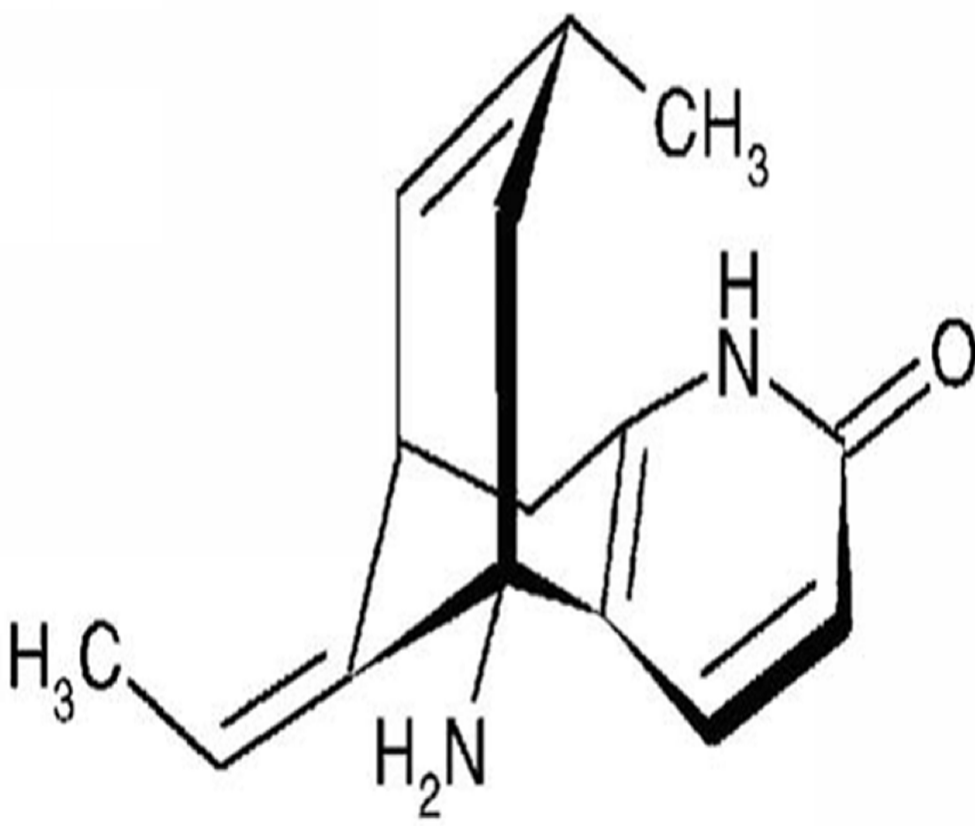
Chemical structure of huperzine A.

Huperzine A, a pyridine-type alkaloid, was first isolated from the traditional Chinese medicinal plant *Huperzia serrata*. The chemical structure of this compound is shown in Fig. 1 [1]. Huperzine A is a highly specific and reversible inhibitor of acetylcholinesterase (AChE) and has low toxicity, thus, it is effective and safe in the treatment of Alzheimer’s disease (AD) [2–4].

*H*. *serrata* produces various types of lycopodium alkaloids, and some of these alkaloids have valuable pharmaceutical applications [5,6]. However, *H*. *serrata* has a limited distribution and a long vegetative cycle. Under native conditions, *H*. *serrata* grows slowly in specific habitats and normally requires 15–20 years to reach maturity after spore germination [7]. Although *H*. *serrata* is the original source of huperzine A, it possesses a low content of huperzine A (80 μg/g cell dry weight) [6]. The considerable health benefits and economic value of *H*. *serrata* place this plant in danger of extinction in China due to extensive harvesting for huperzine A production [6,8]. Therefore, researchers have begun to consider tissue culture for *H*. *serrata* production, but this approach is rarely successful. Although it is possible to synthesize huperzine A chemically [9], the resulting racemic mixture is less potent and has lower AChE inhibition compared with the natural product derived from plant extracts [10–12]. Many studies have isolated huperzine-A-producing endophytic fungi from different Huperiaceae plants, such as *Acremonium* sp. 2F09P03B (huperzine A yielded 8.32 μg/l fermentation broth), *Blastomyces* sp. HA15 (huperzine A yielded 20–30 μg/g dried mycelium), *Botrytis* sp. HA23 (huperzine A yielded 20–30 μg/g dried mycelium), *Shiraia* sp. Slf14 (huperzine A yielded 142.6 μg/g dried mycelium), and *Cladosporium cladosporioides* LF70 (huperzine A yielded 39.61 μg/g dried mycelium) [13–16]. We have isolated a huperzine-A-producing strain, *Colletotrichum gloeosporioides* ES026, from a large variety of endophytes in *H*. *serrata*. The yield of huperzine A from *C*. *gloeosporioides* ES026 extracts reached 45 μg/g dried mycelium [17], indicating that *C*. *gloeosporioides* ES026 is favorable for further study of the biosynthetic pathway of huperzine A.

The biosynthetic pathway of huperzine A remains to be fully elucidated. Only the proposed biosynthetic pathway of huperzine A was reported by Ma and Gang, according to the chemical synthesis of lycopodium alkaloids [5]. In the proposed pathway starting from lysine, cadaverine is catalyzed by lysine decarboxylase (LDC). Cadaverine is further converted to form 5-amiopentanal by a diamine oxidase, and 5-amiopentanal releases molecular water and forms Δ^1^-piperideine. Malonyl-CoA and Δ ^1^-piperideine are further catalyzed to form pelletierine, the first general intermediate of lycopodium alkaloids, by a diamine oxidase, a ketosynthase-type enzyme, or a decarboxylase. Finally, huperzine A is formed through a series of reactions from pelletierine. LDC was annotated and confirmed as the first enzyme to participate in the biosynthesis of huperzine A, and some P450 genes are thought to be involved in the biosynthesis, using high-throughput sequencing of expressed sequence tags (ESTs) from *H*. *serrata* and *Phlegmariurus carinatus* [18,19]. However, these studies were insufficient to fully elucidate the biosynthetic pathway. Compared with the plant genomic data, the fungal genome is simple, and the gene for the key enzyme is more likely to be discovered.

Next-generation sequencing technology, such as deep sequencing-dependent RNA-Seq, especially *de novo* sequencing, is the most widely used strategy for transcriptomic profiling of non-model organisms [1,20]. Among the new next-generation sequencing instruments, HiSeq 2000 is the most inexpensive and has the greatest output, which enables high-throughput RNA-seq. We established a scheme for the common biosynthetic pathway of the huperzine-A-producing endophytic strain ES026 from the transcriptomic data obtained with HiSeq 2000. We predicted several candidate transcripts that may be involved in the biosynthesis of huperzine A. The data analysis and information will contribute to a better understanding of the secondary metabolite biosynthesis of huperzine A, which will further facilitate the developmental regulation and marker-assisted selection of *C*. *gloeosporioides* ES026.

## Materials and Methods

### Strain and Culture Conditions

The endophytic *C*. *gloeosporioides* ES026 strain that produces huperzine A was isolated and screened from *H*. *serrata*. The strain was cultured on Potato Dextrose Agar medium at 25°C in the dark for 7 days. Strain ES026 has been patented in China (Patent Number: ZL 2011 1 005 05 25.2) and is stored at the China Center for Type Culture Collection (CCTCC No.: 2011046; Wuhan, China).

### RNA Isolation and Sequencing

To obtain a complete cDNA libary, the mycelia of *C*. *gloeosporioides* ES026 cultured for 5, 7 and 12 days were collected and immediately mixed and stored in liquid nitrogen until further processing. Total RNA was extracted from 1 g of mycelia using the Total RNA Kit I (Omega Bio-Tek, Norcross, GA, USA) and treated with DNase I for 30 min at 37°C to remove residual DNA. The RNA concentration was measured with a GeneQuant 100 spectrophotometer (GE Healthcare, Chalfont St. Giles, UK). The RNA sample was sent to Bio-Briod (Wuhan Genomic Institute, China) for RNA sequencing. The sequencing was performed with an Illumina HiSeq 2000 (HiSeq 2000 TruSeq SBS Kit v3-HS).

### Sequence Assembly and Analysis

An Illumina PE library (300 bp) was constructed for the application of the Illumina HiSeq 2000 sequencing technology to complete the transcriptome sequencing of *C*. *gloeosporioides* ES026. The raw reads obtained with the Illumina HiSeq 2000 included dirty reads with adapters and unknown or low-quality bases. To perform a more accurate subsequent analysis of the biological information, some raw reads were discarded; this step included removing the linker sequence reads, removing the reads in which the N-containing ratio exceeded 10%, and discarding the adapter. After clean raw RNA-seq data were obtained, the assembly was carried out with a short-reads assembling program; a set of assembled transcript sequences including contigs and unigenes was obtained using Trinity software (http://trinityrnaseq.sourceforge.net/, version number: trinityrnaseq-r2013–02–25), an authoritative software for the *de novo* transcriptome sequence assembly from short-read RNA-Seq data (Grabherr, 2011). Trinity ORF software provided the forecasting process for assembling all the transcripts obtained from the gene sequencing. The sequence assembly of all the transcripts was obtained through the ORF prediction and was divided into the following two parts: predicted ORF (reflected in the protein sequences) and unpredicted ORF (reflected in the nucleotide sequences). The two parts of the sequences were functionally annotated as follows: BLASTX alignment (E value < 1×10^-5^) was compared with the non-redundant protein (NR), Swiss-Prot and Kyoto Encyclopedia of Genes and Genomes (KEGG) databases, and the information with the best match was chosen for the annotation. The direction of the sequence was from the 5′ to the 3′ end. The length of the assembled sequences was standard for a successful assembly.

### Unigene Function Annotation

To find the most likely descriptive annotation for each sequence, the sequence annotation was performed based on a full set of BLAST (BLAST 2.2.25) searches [21].

The NR database of National Center for Biotechnology Information (NCBI) is a comprehensive database, which includes several protein data bank databases, such as Swiss-Prot, PIR (Protein Information Resource), PRF (Protein Research Foundation), PDB (Protein Data Bank) and the translated protein data from GenBank, RefSeq, and CDS. The transcript sequences were aligned to the NR database, and then the sequence information of similar species was chosen for the annotation.

The unigenes were assigned with GO annotation information using the Blast2GO software. The COG (Clusters of Orthologous Groups of proteins, http://www.ncbi.nlm.nih.gov/COG/) database is a protein database in which orthologous gene products are classified. The unigenes were aligned to the COG database to be predicted and classified about their possible functions.

The biochemical pathway assignments were performed based on the KEGG mapping (http://www.genome.ad.jp/kegg/). The database contains a systematic analysis of inner-cell metabolic pathways and the functions of gene products, which contributes to the study of the complicated biological behaviors of genes. The transcript could be given a corresponding KO (KEGG orthology) number through comparison with the KEGG database, and the specific biological pathway of a particular transcript can be acquired according to the KO number.

### Simple Sequence Repeats (SSRs) Detection

The detection of SSRs and design of primers for SSRs from the total unique sequences of *C*. *gloeosporioides* ES026 were performed using Msatcommander software. The software accepts FASTA-formatted sequence files and reports of the sequence ID, SSR motif, number of repeats (di-, tri-, tetra-, penta- or hexa-nucleotide repeat units), repeat length, position of the SSR, and the total length of the sequence in which the SSRs are found [22]. The detection parameters for the maximum motif-length and the minimum number of repeats were set to hexamer and 4, respectively.

### Expression of copper amine oxidase (CAO) gene in *C*. *gloeosporioides* ES026 and *H*. *serrata*

An optimized real-time polymerase chain reaction (PCR) method was used to quantify the expression of CAO gene during the fermentation of *C*. *gloeosporioides* ES026, which is most likely involved in biosynthesis of huperzine A. The mycelia of *C*. *gloeosporioides* ES026 were obtained from 3-, 4-, 5-, 6-, 7- and 8-day-old cultures grown on Potato Dextrose Broth at 25°C with shaking at 150 rpm. Total RNA for real-time quantitative reverse transcriptase PCR (qRT-PCR) was extracted from 100 mg of mycelia with RNeasy Plant Mini Kit (Qiagen Sciences, Hilden, Germany) and purified by treatment with a TURBO DNA Free Kit (Ambion, Austin, TX, USA). Reverse transcription was performed on 1 μg total RNA with the TransScript One-Step gDNA Removal and cDNA Synthesis SuperMix (Beijing TransGen Biotech Co. Ltd. China). Real-time PCR was performed using SYBR Green Real-time PCR Master Mix Plus Kit (Toyobo, Osaka, Japan) with the forward primer 5′-TTTTGTAGGCAACGGGTTTGC-3′ and the reverse primer 5′-CCTCATGGAATCGGCTATGGA-3′. According to the manufacturer’s instructions of the real-time thermal cycler (ABI7500; Life Technologies, Carlsbad, CA, USA), the PCR condition was as follows: denaturation at 94°C for 30 s; 40 cycles of 94°C for 5 s and 55°C for 15 s. The program was followed by a melting-curve program (65–95°C with a 5-s hold at each temperature). The mean value of four replicates was normalized using *α-tubulin* (Unigene 10669) as the internal control with the forward primer 5′-CTTGCTCTTCTTGCCATAGTCG-3′ and the reverse primer 5′-CCTTCAGGGCTTCCTCGTCT-3′. The relative expression levels were calculated using the 2^-ΔΔ CT^ method [23]. Mycelia that had been cultured for 3–8 days were collected and huperzine A was extracted and quantified by HPLC, according to the method of Zhao *et al*. [17].

The expression of CAO gene in *H*. *serrata* plants was also verified used PCR, as described previously but with some modification [27]. *H*. *serrata* plants that attained a height of 10 cm (grown in the wild for 8–10 years) were collected at Fubaoshan (N 30.6°, E 114.3°) at an altitude of 1,450 m in November 2013. The collection was approved by the Science and Technology Bureau of Lichuan city, Hubei province, China. The plants were authenticated by Prof. Qing Wang from Wuhan Botanical Garden, Chinese Academy of Sciences. Total RNA was extracted from 0.1 g leaves, stems and roots using RNeasy Plant Kit (BioTeke, Beijing, China). Reverse transcription was performed with 1 μg total RNA using the Reverse-It First-strand Synthesis Kit (ABgene). The PCR conditions were as follows: denaturation at 94°C for 3 min; 35 cycles at 94°C for 30 s, 55°C for 30 s, and 72°C for 30 s; with a final step of 72°C for 10 min. *α-tubulin* (Unigene 10669) was used as a control. The primers of unigene 9322 and 10669 were as described previously.

## Results

### Generation and assembly of the *de novo* transcriptomic sequencing data

**Fig 2:**
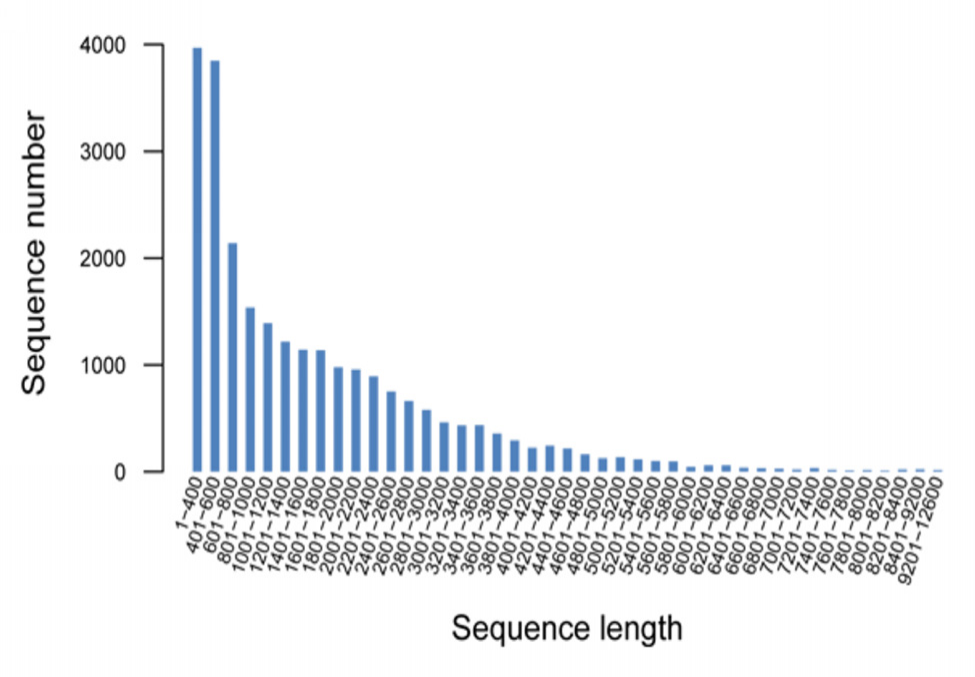
Assembled contig length distribution of *C*. *gloeosporioides* ES026 transcriptome.

To obtain an overview of the gene expression profile of *C*. *gloeosporioides* ES026 during development, cDNA samples from different developmental stages (5, 7 and 12 days) were prepared and sequenced with an Illumina HiSeq 2000 machine. A total of 45,630,118,860 bases from 50,700,132 sequence reads of *C*. *gloeosporioides* ES026 were acquired. A total of 4,324,299,051 bp from 50,442,617 high-quality sequence reads of ES026 were obtained. These raw data were assembled into 24,998 unigenes, 40,536,684 residues and 19,790 genes. The average assembly length was 1,621.6 bp, and the largest unigene was 12,565 bp, whereas the smallest unigene was 301 bp (Table 1). The distribution of the unigene sizes revealed that more than half of the contigs (12,882) were between 200 and 1,200 bp in length (Fig. 2). Moreover, this dataset provided the first available information about the transcriptomes of huperzine-A-producing endophytic fungi. The sequencing data were submitted to Genbank, and the accession number of this project was SRP038110.

**Table 1:** Overview of the sequencing and assembly of ES026.

Description		Number
Sequencing	Total nucleotide (bp)	45,630,118,860
	High-quality nucleotide (bp)	4,324,299,051
	Total sequence	50,700,132
	High-quality sequence	50,442,617
Assembly	Total genes	19,790
	Total unigenes	24,998
	Total residues	40,536,684
	Average length (bp)	1,621.6
	Largest unigene (bp)	12,565
	Smallest unigene (bp)	301

### Annotation of unique putative transcripts

The BLASTX alignments (E value <1×10^-5^) between the predicted protein sequences and several protein databases, including GenBank non-redundant and Swiss-Prot, showed that 16,990 (67.9%) predicted proteins were annotated with known biological functions, whereas the remaining required more biological information in the database.

### Classification of GO and COGs

**Fig 3:**
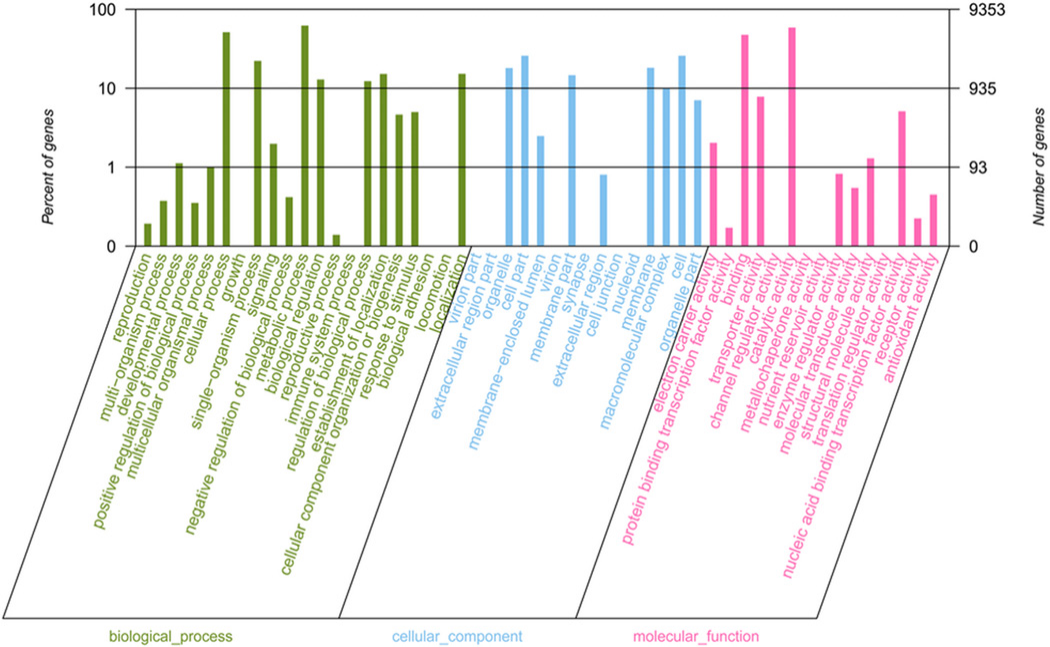
Functional annotation of unique putative transcripts from *C*. *gloeosporioides* ES026 based on GO categories.

Classification of GO was used for the functional categorization of the annotated genes. Based on the biological processes, molecular functions, and cellular components in *C*. *gloeosporioides*, 9,353 of the assembled unigenes were categorized into 51 main functional groups (Fig. 3). A total of 11,671, 11,484, and 19,299 GO terms were assigned to these three major categories. For the category of cellular components, cell parts (2,412, 25.79%) and membrane (1,698, 18.15%) were the most abundant. Catalytic activity (5,493, 58.73%) and binding regulation (4,439, 47.46%) were the maximum number of transcripts in the category of molecular functions. In the category of biological processes, cellular processes (4,786, 51.17%) and metabolic processes (5,808, 62.09%) were predominant.

**Fig 4:**
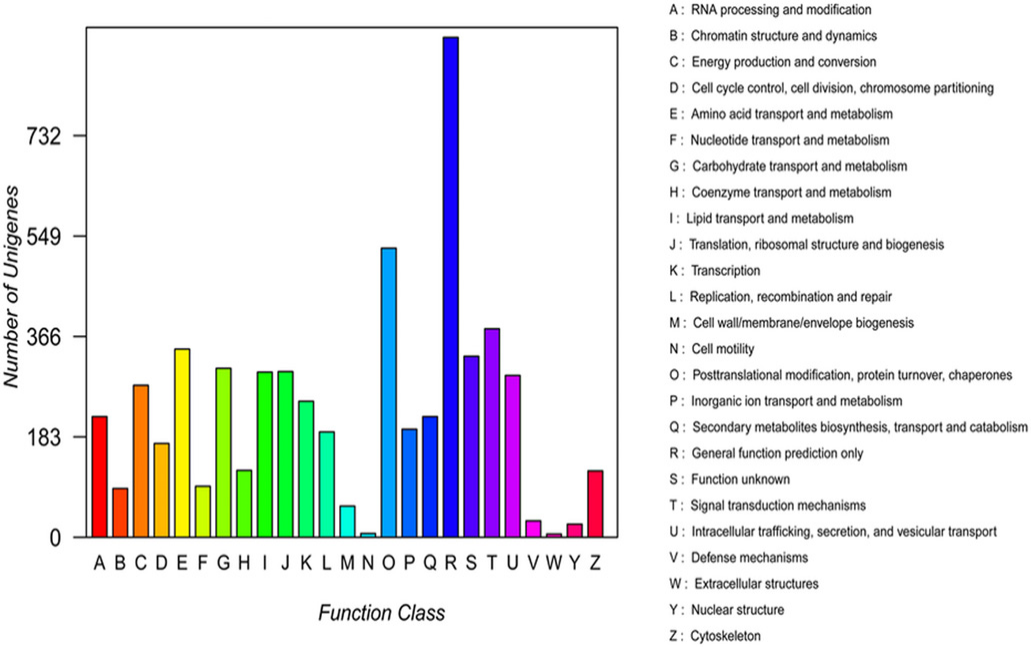
Histogram of COG classification.

To evaluate the completeness of our transcriptomic library and effectiveness of our annotation process, assignments to COGs were used. Overall, 9,353 unigenes were classified as being involved in different processes (Fig. 4). Among the 25 COG categories, most clusters were ‘general function prediction only’ (1,200, 19.28%), ‘translation, ribosomal structure and biogenesis’ (317, 5.09%), ‘carbohydrate transport and metabolism’ (592, 9.51%), ‘transcription’ (310, 4.98%), ‘replication, recombination and repair’ (356, 5.710%), and 2.99% (186) of the clusters were not assigned to any function.

### Detection of SSRs

Microsatellites [simple tandem repeats (STRs) or SSRs] have been used as a resource for random candidate markers in population genetics studies. To better understand the natural diversity of *C*. *gloeosporioides* ES026 and to develop strategies for its sustainable utilization, we identified SSR motifs in our dataset. Some of these SSRs were tightly linked with the functional genes that may control some agronomic characteristics, as well as being involved in the biosynthesis of certain bioactive compounds. Among the SSRs, trinucleotides (2,895) were the most abundant repeat units, followed by mononucleotides (1,886), dinucleotides (754), tetranucleotides (190), pentanucleotides (81) and hexanucleotides (41). Regarding the occurrence of SSRs in *C*. *gloeosporioides* ES026, the trinucleotide repeat units were the most predominant SSR, followed by mono-nucleotide and di- repeat units.

**Fig 5:**
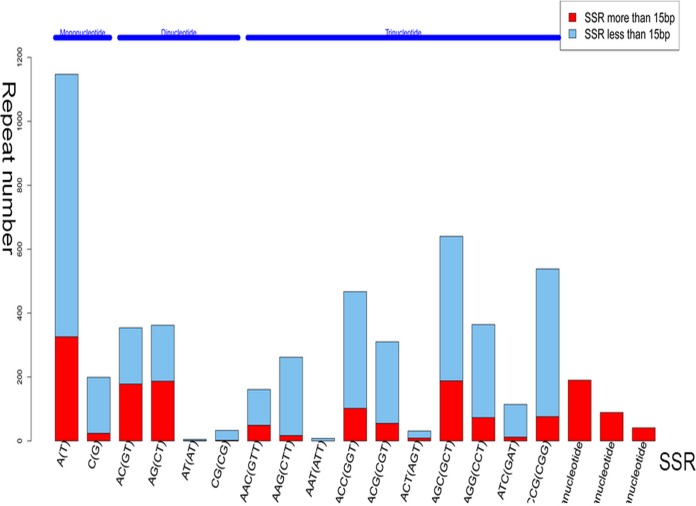
Number of each type of SSR in *C*. *gloeosporioides* ES026.

The relative frequency of the repeats with different dinucleotide compositions was also biased toward one of the four possible repeat classes in *C*. *gloeosporioides* ES026. Among the trinucleotide repeats, AGC (GCT) was the largest repeat class, while AAT (ATT) repeats were infrequent (Fig. 5). Among the dinucleotide repeat classes, AG (CT) repeats were the most common dimer motif, followed by AC (GT) and CG (CG), while AT (AT) repeats were infrequent (Fig. 5). Over 40% of the SSRs were longer than 15 bp (Fig. 5). These unique putative transcript-derived SSR markers generated in the present study provide a valuable genetic resource for future studies of *C*. *gloeosporioides* ES026 and other related endophytic fungi.

### Genes related to biosynthesis of huperzine A

**Fig 6:**
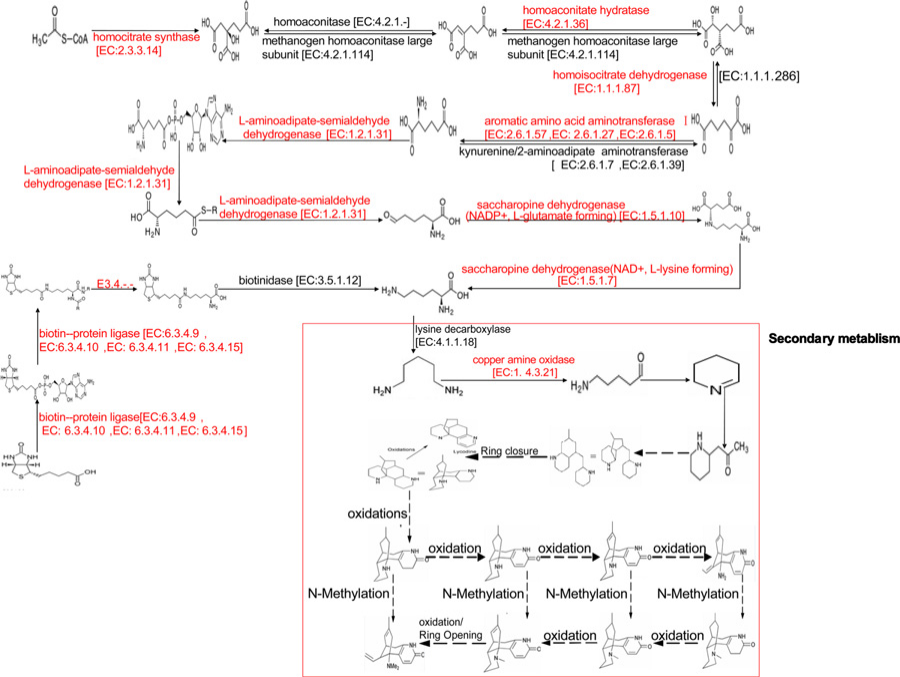
Proposed biosynthetic pathway for huperzine A in *C*. *gloeosporioides* ES026. Enzymes that were annotated in the transcriptome of *C*. *gloeosporioides* ES026 are presented in red, and those that could not be annotated are presented in black. Two arrows used for reversible reaction, and arrow used for irreversible reaction.

Distribution of the gene functions and biochemical pathways in *C*. *gloeosporioides* ES026 was based on the GO and KEGG assignments; particularly in the categories of molecular function and metabolism. These annotations provide valuable resources for investigation of gene functions and cellular structures and processes in *C*. *gloeosporioides* ES026. Three pathways were involved in alkaloid biosynthesis among the 308 metabolic pathways (annotated by KEGG): lysine biosynthesis; biotin metabolism; and tropane, piperidine and pyridine alkaloid biosynthesis. A total of 30 unigenes in this library showed similarities to the uncharacterized enzymes that might be associated with the biosynthesis of huperzine A (Table 2). Based on these three pathways and the research of Ma and Gang, a more detailed biosynthetic pathway was deduced (Fig. 6) and the enzymes and genes in Table 2 were annotated in the pathway. The discovery of new transcripts will facilitate the elucidation of the biosynthetic mechanisms of huperzine A at the molecular level in *C*. *gloeosporioides* ES026.

**Table 2:** Possible unigenes and encoding enzymes involved in huperzine A biosynthetic pathway.

Pathway	Enzyme	Unigene
Lysine biosynthesis	homocitrate synthase [EC:2.3.3.14]	Unigene15734_ c0_seq1
	homoaconitate hydratase [EC:4.2.1.36]	Unigene 11242_c0_seq1
	homoisocitrate dehydrogenase [EC:1.1.1.87]	Unigene17283_c0_seq1
	aromatic amino acid aminotransferase I [EC:2.6.1.57]	Unigene 14978_c1_seq1
	L-aminoadipate-semialdehyde dehydrogenase [EC:1.2.1.31]	Unigene 9165_c0_seq1
	saccharopine dehydrogenase (NADP+, L-glutamate forming) [EC:1.5.1.10]	Unigene14707_c0_seq1
	saccharopine dehydrogenase (NAD+, L-lysine forming) [EC:1.5.1.7]	Unigene6171_c0_seq1
Biotin metabolism	biotin—protein ligase [EC:6.3.4.9, EC: 6.3.4.10, EC: 6.3.4.11, EC: 6.3.4.15]	Unigene15126_c1_seq1, Unigene15126_c1_seq2, Unigene15126_c1_seq3, Unigene15126_c1_seq4, Unigene15126_c1_seq5
	EC: 3.4.-.- (hydrolysis enzymes acting on peptides bonds)	Unigene17901_c0_seq1, Unigene 9788_c0_seq1
Tropane piperidine, pyridine alkaloid biosynthesis	primary-amine oxidase [EC:1.4.3.21]	Unigene10060_c0_seq1, Unigene10314_c0_seq1, Unigene112700_c0_seq1, Unigene12610_c0_seq1, Unigene12610_c1_seq1, Unigene12610_c2_seq1, Unigene12610_c2_seq2, Unigene13099_c0_seq1, Unigene13099_c1_seq1, Unigene14974_c0_seq1 Unigene16440_c0_seq1, Unigene49225_c0_seq1, Unigene5254_c0_seq1, Unigene8472_c0_seq1, Unigene9217_c0_seq1, Unigene9322_c0_seq1

### Expression of CAO gene in *C*. *gloeosporioides* ES026 and *H*. *serrata*

**Fig 7:**
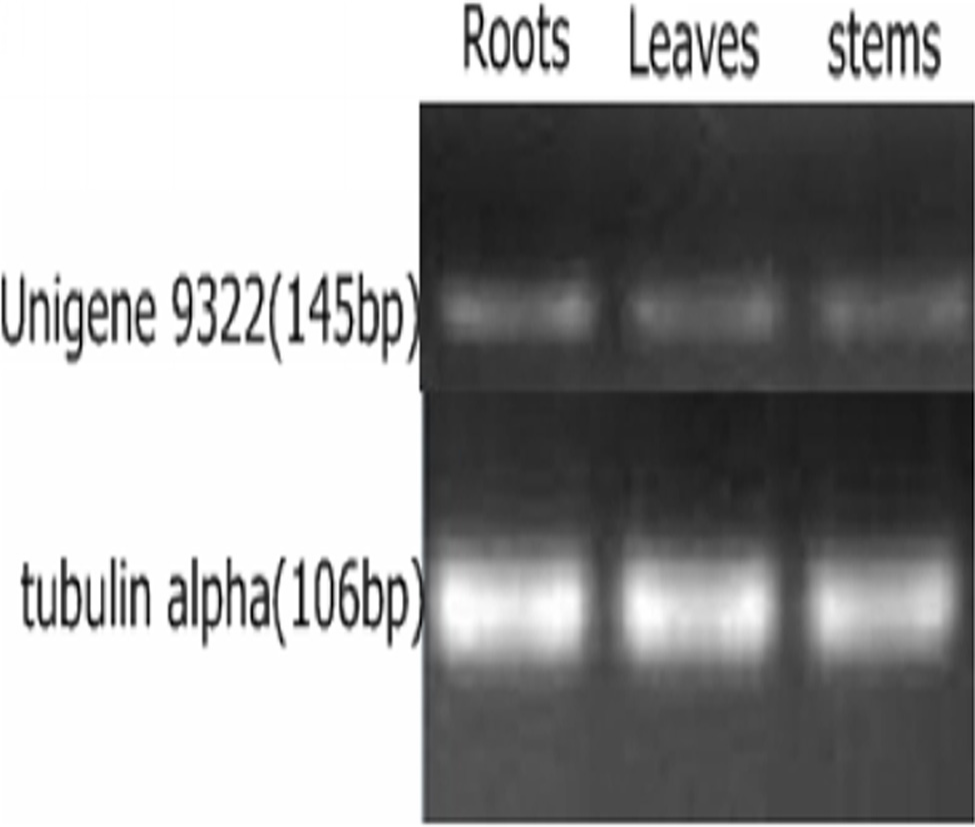
Expression pattern of unigene 9322. Reverse transcription-PCR of unigene 9322 mRNA levels in roots, stems and leaves of *H*. *serrata*, with *α-tubulin* as an internal control.

**Fig 8:**
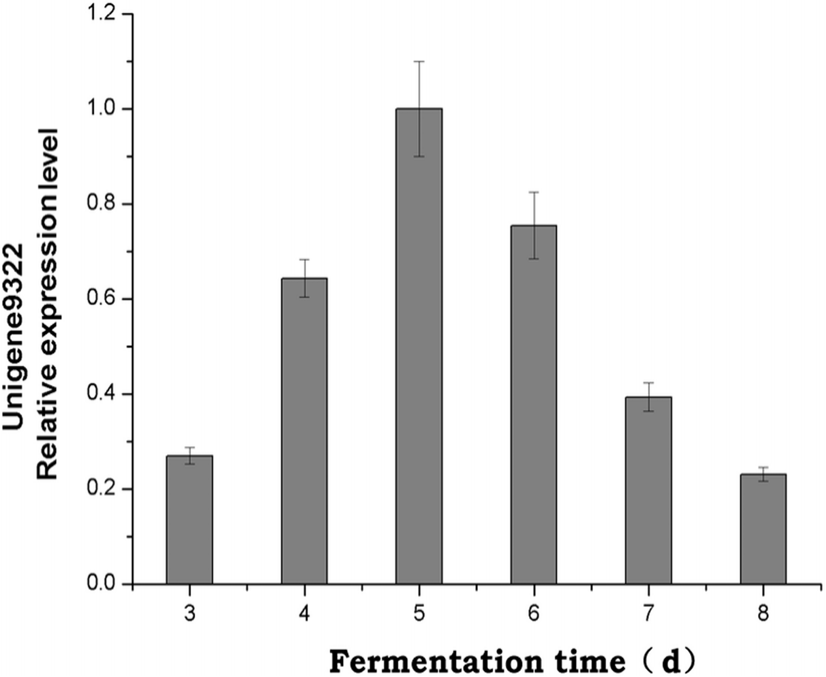
Expression of unigene 9322 from day 3 to 8 in *C*. *gloeosporioides* ES026 fermentation. Values are mean ± SD for four individual replicates.

**Fig 9:**
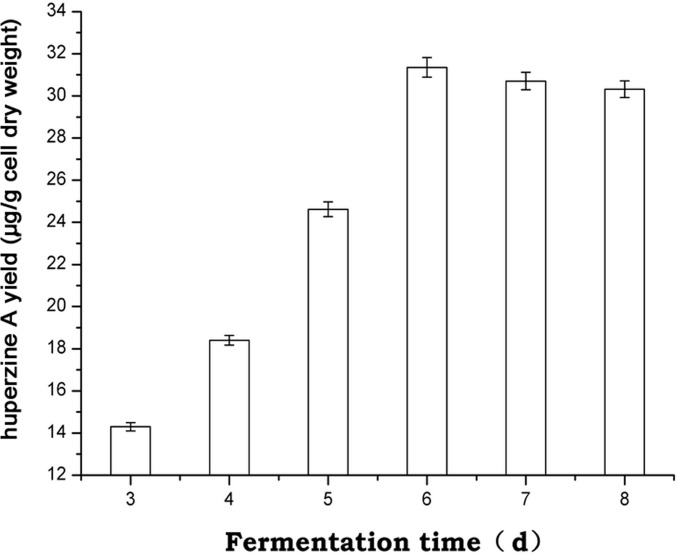
Effect of fermentation duration on huperzine A yield. Values are mean ± standard deviation (SD) for three individual replicates.

Huperzine A originates from the coupling of the pelletierine and the 4PAA (4-(2-piperdyl) acetoacetate) /4PAACoA pathways. In the tropane, piperidine and pyridine alkaloid biosynthesis pathways, CAO (unigene 9322) was annotated for the conversion of cadaverine to 5-aminopentanal in the biosynthesis of huperzine A. Therefore, unigene 9322 was selected for the subsequent PCR analysis of *C*. *gloeosporioides* ES026 and *H*. *serrata*. Unigene 9322 was detected in the roots, stems and leaves of *H*. *serrata* (Fig. 7). At the same time, we found that during *C*. *gloeosporioides* ES026 fermentation, the highest level of expression was from day 3 to 8 (Fig. 8); correspondingly, the highest huperzine A content was detected with HPLC on day 6 (Fig. 9).

## Discussion

*C*. *gloeosporioides* is a plant pathogenic fungus. However, in the present study, strain ES026 was isolated from the stem of *H*. *serrata* and identified as an endophyte that produced huperzine A. Other recent studies have suggested that endophyte—host plant interactions range from antagonistic to mutualistic [24]. Due to long-term growth in the stem of *H*. *serrata*, the pathogenicity of strain ES026 might be reduced or even lost. Finally, a mutualism was formed between strain ES026 and *H*. *serrata*. Regarding long-term mutualism, the close relationship and complex interaction between plant and fungus, endophytes and host plants can produce the same or similar metabolites [25]. Strain ES026 was isolated and verified to produce huperzine A. This provides an optimal material for the study of huperzine A biosynthesis and fermentation production of huperzine A.

We performed RNA sequencing of the huperzine-A-producing endophytic fungus strain ES026 by Illumina HiSeq 2000. These transcriptomic data will improve our understanding of the growth, development and secondary metabolite biosynthesis in *C*. *gloeosporioides*, thus promoting the developmental regulation of this fungus. Although endophytic fungi offer an excellent opportunity to produce chemicals without the limitation of plant resources, there are critical obstacles such as low and unstable production of metabolites that must be overcome to maintain fungal viability and stability. Therefore, the dataset will provide a significant resource for gene identification and facilitate the enhancement of the vitality and stability of the cells during passage.

On the basis of the transcriptomic data and the researches of Ma and Luo [5,18,19], the biosynthetic pathway of huperzine A was further complemented (Fig. 6). The biosynthesis of huperzine A includes primary and secondary metabolites. Primary metabolism begins from acetyl-CoA and biotin and ends with the formation of l-lysine, while secondary metabolism starts from production of cadaverine. Subsequently, a CAO catalyzes cadaverine to form 5-aminopentanal, and then piperideine and pelletierine are successively synthesized. The pelletierine that acts as a precursor is further catalyzed, which leads to synthesis of huperzine A. In the pathway, LDC is the first enzyme in the secondary metabolism of huperzine A. LDC participates in the transformation of l-lysine to cadaverine in lycopodium alkaloid biosynthesis [26]. A unique sequence (GO914645) encoding a full-length *LDC*-like gene with an unknown function was found in the EST collection and was named as *HsLDC* [18,19]. From the tropane, piperidine and pyridine alkaloid biosynthesis pathway in the KEGG database, there is only one pathway to synthesize cadaverine that is catalyzed by LDC. However, we could not annotate this enzyme in our transcriptomic data. There may be other unknown enzymes or pathways to synthesize cadaverine.

In the KEGG pathway database, CAO was annotated for the conversion of cadaverine to 5-aminopentanal. A CAO (EC 1.4.3.21) gene has been cloned and sequenced from the roots of *H*. *serrata* (Huperziaceae) by Sun and Morita et al [27]. Full-length cDNA contained a 2,043-bp ORF encoding a 76,854 MW protein with 681 amino acids (GenBank ID: JN247732). CAO can catalyze cadaverine for oxidative deamination to produce Δ^1^-piperideine. In our study, this gene was also annotated and detected in roots, leaves and stems of *H*. *serrata* using PCR. Thus, CAO might be an enzyme involved in the biosynthetic pathway of huperzine A.

CAO gene expression and yield of huperzine A during fermentation of strain ES026 were observed. During quantification of CAO gene expression, the qRT-PCR conditions were optimized for the fungus only, and a two-step qRT-PCR was used. The melting curve (data not shown) with a single peak indicated that the PCR was specific and efficient. The relative expression of CAO gene increased gradually, reached a maximum on day 5 and then started to decrease. The yield of huperzine A increased correspondingly and reached a maximum on day 6. In general, the protein function appeared later than gene expression, because there are some processes including protein translation, post-translational modification, and protein transfer, between gene expression and enzymatic catalysis. On day 6, the access of huperzine A could have negative regulatory role for the expression of CAO gene, so the gene expression began to decrease from that day. Due to accumulation of huperzine A, gene expression was decreased between days 6 and 8, while the huperzine A levels remained high during the same period. There might be a positive correlation between expression of CAO gene and the contents of huperzine A during fermentation of *C*. *gloeosporioides* ES026. Thus, CAO might be a race-limiting enzyme that regulates huperzine A biosynthesis in *C*. *gloeosporioides* ES026. However, the regulatory effect of CAO on huperzine A biosynthesis needs to be verified using feeding studies and CAO gene overexpression and RNA interference.

P450 genes were thought to be required for the biosynthesis of huperzine A, but none was confirmed [19]. The transcriptome of *C*. *gloeosporioides* ES026 revealed that ≥200 putative cytochrome P450 genes were sequenced. Accordingly, no cytochrome P450 gene was annotated in the biosynthetic pathway of huperzine A. This shows that there are many genes involved in the biosynthesis pathway of huperzine A that remain to be discovered, and many unknown genes waiting for annotation in our transcriptomic data.

Although we have not fully revealed the biosynthesis pathway of huperzine A, we have further elucidated it and obtained some additional candidate genes. In the future research, we could identify the catalytic enzymes according to the chemical structures of the materials in the biosynthesis pathway of huperzine A. Hence, the putative genes that encode these enzymes can be identified from the transcriptome of *C*. *gloeosporioides* ES026, and their functions can be verified through biological experiments such as RNA interference, gene knock-out, and gene overexpression. This strategy will contribute to complete elucidation of the biosynthesis of huperzine A. With the development of synthetic biology, the biosynthesis pathway of huperzine A can be assembled in other microorganisms that are easily genetically manipulated, which can increase the yield of huperzine A. Furthermore, the *de novo* sequencing utilized in this study is highly recommended for elucidating the biosynthetic pathways for the production of natural bioactive products, which will provide a new incentive for the discovery of specific genes and pathway-based studies.
